# Study on Temperature Response of Rubberized Concrete Pavement Based on Fiber Bragg Grating Testing Technology

**DOI:** 10.3390/s24175545

**Published:** 2024-08-27

**Authors:** Gaojun Zhang, Gaowang Zhang, Jie Yuan, Manman Su

**Affiliations:** 1 Lanzhou New Area Urban Construction Engineering Co., Ltd., Lanzhou 730087, China; 18209217335@163.com; 2 School of Civil and Architecture Engineering, Xi’an Technological University, Xi’an 710021, China; 3 Key Laboratory of Road and Traffic Engineering of the Ministry of Education, Tongji University, Shanghai 201804, China; 4 School of Civil Engineering, Yantai University, Yantai 264005, China; suman0725@ytu.edu.cn

**Keywords:** pavement engineering, rubberized cement concrete, temperature field, strain and stress, fiber Bragg grating sensor

## Abstract

The temperature response of pavement is not only crucial for assessing the internal stresses within pavement structures but is also an essential parameter in pavement design. Investigating the temperature response of rubberized concrete pavements (RCP) can support the construction of large-scale rubber concrete pavements. This study constructed a pavement monitoring system based on fiber Bragg grating technology to investigate the temperature distribution, temperature strain, temperature effects, and temperature stress of RCP. The results show that the daily temperature–time history curves of concrete pavement exhibit a significant asymmetry, with the heating phase accounting for only one-third of the curve. The temperature at the middle of RCP is 1.8 °C higher than that of ordinary concrete pavement (OCP). The temperature distribution along the thickness of the pavement follows a “spindle-shaped” pattern, with higher temperatures in the center and lower temperatures at the ends. Additionally, the addition of rubber aggregates increases the temperature strain in the pavements, makes the temperature–strain hysteresis effect more pronounced, and increases the curvature of the pavement slab. However, the daily stress range at the bottom of RCP is approximately 0.7 times that of OCP.

## 1. Introduction

Concrete pavements are characterized by high strength and long service life, making them the primary structural form for airport runways or heavy load pavements. With the developments of cement materials and the large-scale construction of concrete pavements, extensive research has been conducted on the performances of pavement materials and response of concrete pavement structures [1,2,3,4,5]. In terms of the pavement responses, plenty of researchers have found that the temperature distribution and temperature strain of concrete pavements are uneven due to environmental temperatures, solar radiation, and other factors. Those uneven distributions can easily lead to upward or downward curling of the pavement, which negatively impacts its performance and service life [6,7].

To date, many scholars have conducted numerous studies on the temperature distribution and temperature effects of concrete pavements using theoretical and empirical methods. The description of temperature fields has progressed from initially linear models to more complex nonlinear models. Based on these findings, Nantasai [8] established different types of pavement temperature prediction models and validated the prediction models through measured data, demonstrating their broad applicability. Liang [9] proposed an analytical solution for temperature distribution in concrete pavements and calculated bending stress and moment based on thermal analysis and Winkler’s plate theory of foundation. In recent years, with the development of pavement monitoring technology, researchers were able to obtain the temperature strain and stress of the pavement more accurately. Zhang [10], Quan [11], and Liao [12] found that the horizontal distribution of temperature effects in concrete pavements was non-uniform by monitoring temperature and strain at different positions and depths of the concrete pavement. These research findings have enhanced the understanding of the temperature characteristics in concrete pavements and provide valuable support for the design and stress calculations of concrete pavements. Therefore, it is of great significance to clarify the temperature response of concrete pavement.

Rubberized cement concrete is an innovative material created by substituting a certain volume of aggregates with rubber particles in cement concrete. Due to the excellent elasticity, toughness, seismic resistance, and damping properties of rubber particles, adding them into concrete can effectively improve the toughness, impact resistance, and fatigue resistance of the concrete [13,14,15,16]. Furthermore, rubber particles can absorb various stresses generated in concrete, inhibit concrete shrinkage deformation, and prevent or slow down concrete cracking caused by microcracks or shrinkage deformation. Therefore, Zhu [17] and Zhang [18] have proposed a concept for constructing large-scale concrete pavements using rubberized cement concrete. These large-scale concrete pavements can effectively solve the distresses faced by the common concrete pavements, such as freeze–thaw cracking, corner breakage, and failure of joint fillers. However, the addition of rubber particles alters the aggregate composition, mixing proportions, and thermodynamic parameters of the concrete, thereby impacting the temperature field of rubberized concrete pavement (RCP). Sukontasukkul [19] and Ocholi [20] tested the effect of rubber aggregates on the thermal properties of concrete and found that replacing some mineral aggregates with rubber particles resulted in a reduction in thermal diffusivity, thermal conductivity, and heat capacity of the concrete. Chen [21] found that the factors affecting the temperature distribution of pavement included not only the climate data but also the thermal performance parameters of pavement materials. When calculating the energy exchange of pavement with the empirical formulas, it was suggested to calibrate these formulas according to the thermal performance parameters of different materials. Meanwhile, through numerical simulations and theoretical analysis, Xue [22] and Ferretti [23] found that the addition of rubber particles altered the stress distribution at the bottom of concrete pavement, reducing the stress at the middle of the transverse and longitudinal joint edge. In conclusion, compared to ordinary concrete pavement (OCP), the addition of rubber particles not only alters the temperature distribution of concrete pavements but also affects the modulus of concrete. This, in turn, leads to different stress and strain responses under the same load conditions. So, it is essential to study the temperature response of RCP.

Meanwhile, with technological advancements, new testing technologies have provided convenience for testing the temperature responses of concrete pavements compared to the traditional testing technologies, with fiber Bragg grating technology being the most representative [24,25]. Fiber Bragg grating sensors are highly sensitive, precise, resistant to corrosion and water, and immune to electromagnetic interference, so they are the preferred sensors for intelligent monitoring systems, especially suitable for concrete environments with high water and high alkali content. Wang [26] reviewed the application of the fiber Bragg grating sensors in pavement monitoring and highlighted that the fiber Bragg grating sensors could accurately monitor the parameters of pavement such as strain, temperature, and pressure, but also warned that the attention should be paid to sensor protection during construction to ensure survival rates. Zhou [27] established a long-term monitoring system for concrete pavement performances using fiber Bragg grating sensors and demonstrated that fiber Bragg grating technology offered a promising solution for pavement health assessment. Kashaganova [28] employed a monitoring system with fiber optic grating sensors to accurately monitor the temperature and deformation of concrete pavement, providing crucial information for assessing the pavement service life. Thus, monitoring the temperature responses of RCP based on fiber Bragg grating sensors can provide engineers and researchers with accurate and precise, real-time temperature response data, providing effective assistance in design and evaluation.

## 2. Objectives and Scope

In summary, understanding the temperature response of pavement is very important in the study of pavement structure. This response is not only a prerequisite for determining the internal stress and strain of the pavement structure but is also a significant parameter in concrete pavement design. At present, the research on RCP has mainly focused on the material characteristics of rubberized concrete and numerical simulation of the pavement structure. However, there was a paucity of research on the temperature response of RCP, with a lack of empirical data from the actual engineering project, which limits support for the design and construction of large-scale RCP.

Therefore, this paper aims to study the temperature responses of RCP. Firstly, the long-term temperature response monitoring system of concrete pavement was established based on fiber Bragg grating sensors. Then, the differences of temperature field distribution and temperature strain between OCP and RCP were compared and analyzed by using the data obtained from the monitoring system, respectively. At the same time, the relationships between temperature and strain were analyzed, and the influences of rubber aggregates on the temperature expansion–contraction effect and temperature curling effect of concrete pavement were discussed. Finally, the temperature stresses in RCP and OCP were calculated and compared based on the elastic plate theory.

Through this study, the temperature responses of RCP and the differences in temperature field, temperature strain, and temperature stress between RCP and OCP can be clarified. It not only further demonstrates the feasibility of constructing large-scale RCP but also provides valuable on-site data for the design and construction of large-scale RCP.

## 3. Materials and Experiment Procedure

### 3.1. Materials

Rubberized cement concrete was produced by replacing sand with rubber aggregates on a volume basis. Table 1 shows the technical characteristics of rubber aggregates. The mixing proportions of rubberized cement concrete and ordinary cement concrete are shown in Table 2. The water–cement ratio of concrete is set at 0.40, with a water-reducing agent to adjust the workability of the concrete. Concrete specimens for mechanical property tests were prepared from concrete mixed on-site during construction. The mechanical property tests were conducted according to the specifications of JTG 3420-2020, including compressive strength, flexural strength, and elasticity modulus of concrete. The specimen dimensions were 150 mm × 150 mm × 150 mm for compressive strength, 150 mm × 150 mm × 550 mm for flexural strength, and 150 mm × 150 mm × 400 mm for modulus of elasticity [29]. The specimens were cured in water at the temperature of 25 ± 2 °C for 28 d. the experimental results are shown in Table 3.

### 3.2. Pavement Structure

**Figure 1:**
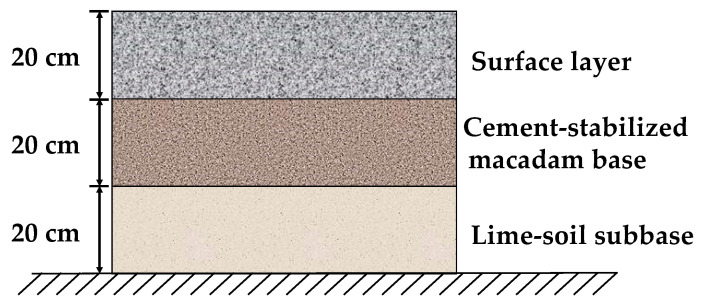
Structure of concrete pavement.

The study was conducted on a specific pavement project in China, where test sections for both RCP and OCP were established, as depicted in Figure 1. Both RCP and OCP had identical base and subbase thicknesses and materials: a 20 cm thick base of cement stabilized macadam and a 20 cm thick subbase of lime soil. The experimental pavement area measured 12 m in length and 4 m in width. The sections lacked dowel bars, and tie bars were placed only longitudinally, following the direction of traffic flow. In order to reduce the impact of loads on the pavement’s temperature response, the test area was located at the end area of the project site.

### 3.3. Sensor Parameters and Layout Scheme

The study utilized fiber Bragg grating temperature and strain sensors to monitor the pavement temperature response, the technical parameters of which are shown in Table 4.

**Figure 2:**
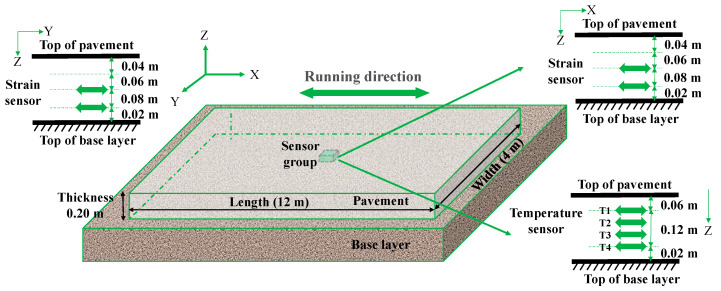
Schematic diagram of sensor layout.

To ensure the sensors accurately reflect the temperature and strain of the concrete pavement, it is crucial for them to bond effectively with the concrete. Therefore, the study employed a construction method where sensors were installed before pouring the pavement concrete. This approach minimizes the risk of poor bonding between the concrete and the sensors, ensuring data accuracy. Specifically, after completing the construction of the cement-stabilized macadam base layer, steel supports were placed on the base layer at the designed positions as shown in Figure 2. Subsequently, fiber Bragg grating temperature and strain sensors were fixed on these steel supports using the tie bands. Throughout the installation process, a steel ruler was employed to continuously verify the sensors distance to ensure they met specifications. Finally, the surface layer concrete was poured, during which manual vibration techniques were employed in close proximity to the sensors to prevent any displacement or misalignment.

**Figure 3:**
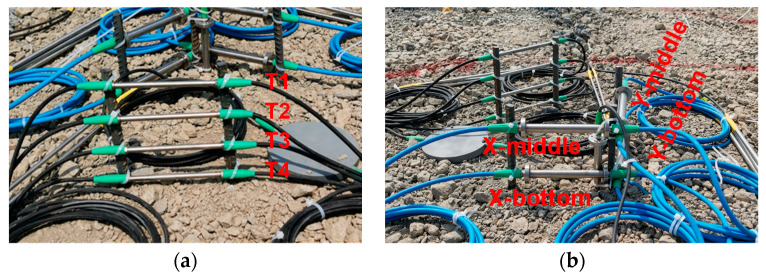
Installation of sensors: (**a**) temperature sensors; (**b**) strain sensors.

The fiber Bragg grating sensor groups were positioned in the center of the pavement, with each group consisting of four strain sensors and four temperature sensors. The detailed sensor placement is shown in Figure 2 and Figure 3, where the *x*-axis, *y*-axis, and *z*-axis denote the longitudinal, transverse, and vertical directions of the pavement, respectively, corresponding to the directions of vehicle running, pavement width, and pavement thickness. In order to minimize the impact of construction on the sensors, safety clearances were established: 0.06 m from the pavement slab surface for the uppermost sensors and 0.02 m from the pavement slab bottom for the lowermost sensors during installation. The four temperature sensors were evenly spaced along the *z*-axis at distances of 0.06 m, 0.10 m, 0.14 m, and 0.18 m from the pavement surface, designated as T1, T2, T3, and T4, respectively. These sensors were used to monitor the temperature distribution along the thickness direction of the concrete pavements. Along the *x*-and *y*-axes, two strain sensors were placed at the middle and bottom of the pavement, located 0.10 m and 0.18 m from the pavement surface, respectively. These sensors were used to monitor temperature strain horizontally in the middle and bottom of the concrete pavement. For ease of the subsequent description and analysis, the strain sensors were designated as X-bottom, X-middle, Y-bottom, and Y-middle, according their positions and directions.

### 3.4. Experiment Procedure

A fiber Bragg grating demodulator with wireless transmission capability was used for data acquisition, whose technical parameters are shown in the Table 5. All sensors were connected to the demodulator by optical cables, and the demodulator was powered by photovoltaic technology, thereby establishing a real-time monitoring system for concrete pavements. The data acquisition interval of the demodulator was set to 10 min, and the monitoring continued for 30 days. Data collected during periods of clear weather without precipitation were selected for temperature response analysis to minimize the influence of humidity on the results.

During operation, the demodulator collected the wavelength information from both the fiber Bragg grating strain sensors and temperature sensors. Meanwhile, the demodulator converted the obtained wavelength changes form the strain sensors into strain values according to Equation (1) and converted the obtained wavelength changes from the temperature sensors into temperature values according to Equation (2).

(1) $$S=K_{S}\times \left[\left(\lambda_{S}-\lambda_{0}\right)-K_{T}^{\prime }\times \left(\lambda_{T}-\lambda_{T0}\right)\right]$$

where *S* is the measured strain, με; *K_S_* and $K_{T}^{\prime }$ are the strain sensitivity coefficient (με/nm) and temperature compensation coefficient, respectively, which are fixed parameters of the sensor; *λ_S_*, *λ*_0_, *λ*_T_ and *λ*_T0_ are the measuring wavelength of the strain grating, initial wavelength of the strain grating, measuring wavelength of the temperature grating, and initial wavelength of the temperature grating, nm.

(2) $$T=K_{T}\times \left(\lambda_{t}-\lambda_{0}\right)+T_{0}$$

where *T* is the temperature, °C; *K*_T_ and *T*_0_ are, respectively, the temperature sensitivity coefficient of the sensor and constant of the sensor, which were provided by the sensor manufacturer; *λ*_t_ and *λ*_0_ are the test wavelength and initial wavelength, nm.

## 4. Results and Discussion

### 4.1. Temperature Field

To study the daily temperature field of RCP and OCP, the data (72 h) with the most typical temperature and the largest temperature variation during the monitoring period were selected as examples. The former represents a normal temperature period, while the latter represents a cooling period. Through these examples, the temperature distribution and gradients of the concrete pavement were analyzed.

#### 4.1.1. Temperature Distribution

**Figure 4:**
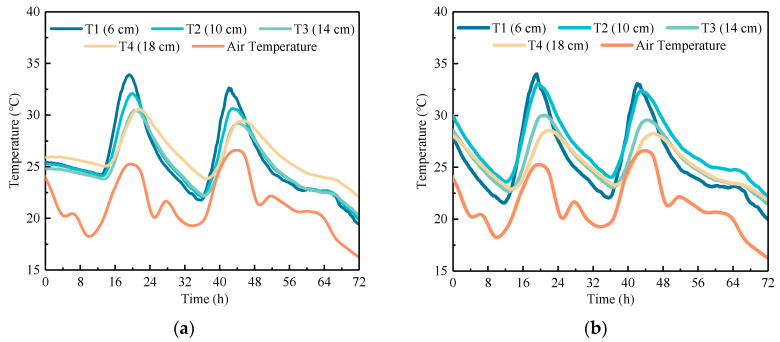
Temperature–time history curve of concrete pavement under normal temperature conditions: (**a**) OCP and (**b**) RCP.

**Figure 5:**
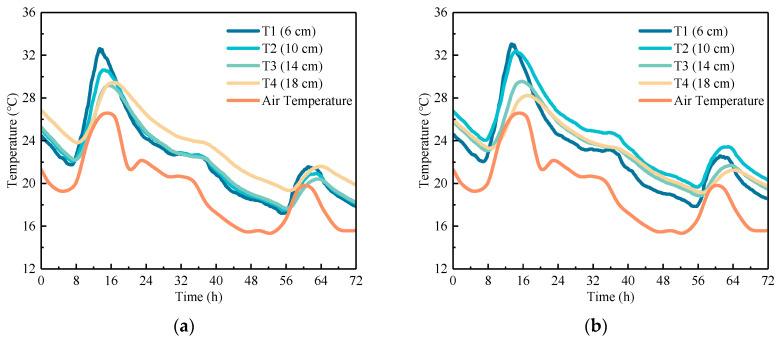
Temperature–time history curve of concrete pavement under cooling conditions: (**a**) OCP and (**b**) RCP.

The temperature field of concrete pavement during its service life is mainly influenced by environmental factors and heat exchange. Figure 4 and Figure 5 present the temperature–time history curves along various depths within the pavement center. It can be observed that these curves have a significant cyclic variation in pavement temperature, mirroring air temperature fluctuations. During the heating phase, the top of the slab heats up most rapidly, with a noticeable delay in temperature rise at greater depths. In detail, the temperature at the bottom of OCP lags the top by about 2.5 h, while for RCP, the lag is approximately 3 h. The cooling phase follows a similar pattern to the heating phase. The minor fluctuations in air temperature have a negligible effect on the pavement temperature. Within a single temperature cycle (24 h), the heating phase of the concrete pavement accounts for one-third of the temperature change, while the cooling phase accounts for two-thirds, resulting in a distinct asymmetry in the daily temperature variation curve of the concrete pavement. Meanwhile, under normal temperature conditions, it can be found that the cooling rates at the top and middle of OCP slab are greater than that at the bottom of the slab, while for RCP, the cooling rates at the top and bottom of the slab are greater than the rate at the middle. Under cooling temperature conditions, the effect of the temperature at the middle of the slab being higher than the top and bottom temperatures becomes more pronounced. At 30 h, the temperature at the middle of RCP slab is 1.98 °C higher than that at the top and 0.85 °C higher than that at the bottom. Additionally, the addition of rubber aggregates reduces the sensitivity of concrete to temperature changes, resulting in a delayed temperature response of RCP compared to OCP during both cooling and heating phases.

**Figure 6:**
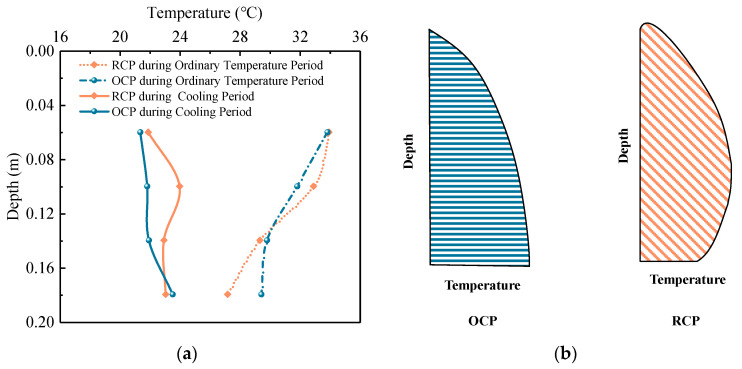
Temperature characteristics of concrete pavement: (**a**) temperature–depth distribution characteristics at different time points and (**b**) temperature field diagram of concrete pavement.

The temperature distributions at the high-temperature moments under the ordinary temperature condition and large temperature change moment under the cooling condition were, respectively, taken to plot the temperature–depth characteristic curves of the concrete pavement, as shown in Figure 6. The figure reveals that the temperature distribution of the concrete pavement varies nonlinearly with depth. Under the normal temperature condition, the temperatures at the top of both RCP and OCP are similar, but the temperature at the bottom of the RCP is lower than that of OCP. During cooling, the RCP exhibits a distinct characteristic where the temperature at the middle of the slab is higher than the temperatures at the top and bottom of the slab, with the temperature at the middle of RCP being 2.1 °C higher than that of OCP. In other words, the temperature distribution of RCP exhibits a unique “spindle-shaped” pattern with higher temperature at the middle and lower temperatures at the ends, while the temperature distribution of OCP displays a “wedge-like” pattern with one end higher than the other. From the perspective of influencing pavement curling, the pattern of RCP will significantly reduce the curling deformation of the RCP, thereby benefiting the pavement in resisting more severe coupling effects of thermal shrinkage and curling effects.

#### 4.1.2. Temperature Gradient

In order to assess the influence of uneven temperature distribution on pavement, the most intuitive way is to calculate the temperature gradient of concrete pavement, which is essential for calculating its curling deformation. For nonlinear temperature distributions, Janssen [30] proposed the concept of temperature moments to describe the equivalent temperature gradients, though this required a precise temperature distribution along the thickness. In this study, detailed temperature distribution curves could not be obtained due to limitations in monitoring, so the temperature gradient was still calculated by Equation (3). In pavement monitoring, to ensure that the sensor did not affect the pavement performance and safety, the top temperature sensor was positioned 6 cm away from the surface of the slab. Therefore, the temperature gradient of concrete pavement was calculated by taking the sensor data at 6 cm and 18 cm as the temperatures at the top and bottom of the slab, respectively.

(3) $$T_{g}=\frac{T_{t}-T_{b}}{Z_{t}-Z_{b}}$$

where *T*_g_ is the temperature gradient, °C/m; *T*_t_ is the temperature at the top of the slab, °C; *T*_b_ is the temperature at the bottom of the slab, °C; *Z*_t_ is the depth of the temperature sensor at the top of the slab, m; and *Z*_b_ is the depth of the temperature sensor at the bottom of the slab, m.

**Figure 7:**
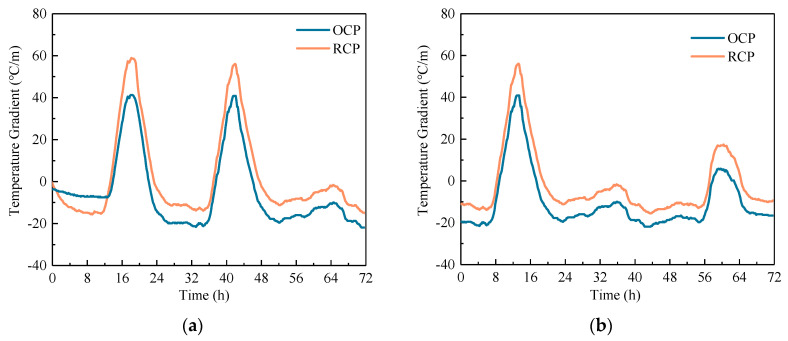
Temperature gradient time history curve of concrete pavement: (**a**) ordinary temperature period and (**b**) cooling period.

Figure 7 shows the temperature gradient time history curves of concrete pavements under both ordinary temperature and cooling conditions. The data show that, under the normal temperature or cooling conditions, RCP experiences higher positive temperature gradients compared to OCP, with the maximum positive temperature gradients being approximately 1.40 times those of OCP. Conversely, RCP experiences smaller negative temperature gradients compared to OCP, with the maximum negative temperature gradients being only 0.66 times those of OCP. This indicates that RCP is more susceptible to downward curling due to higher positive temperature gradients, while the upward curling effects from negative gradients are significantly lower than those of OCP. These factors should be given special consideration in the design and construction of RCP. Additionally, since the negative temperature gradients mostly occur during the low-temperature stage of a temperature cycle, the smaller negative temperature gradients will help RCP resist cracking in low-temperature environments.

#### 4.1.3. Statistical Distribution of Temperature Field

**Figure 8:**
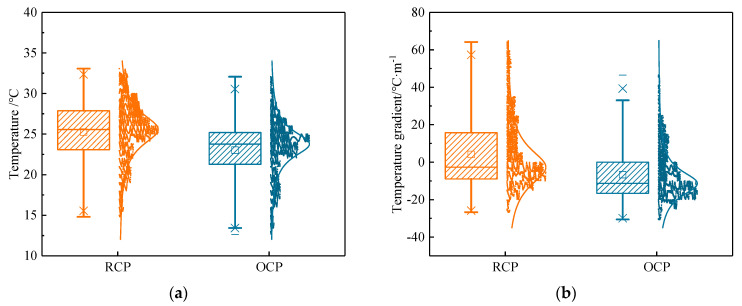
Temperature and temperature gradient statistical results of concrete pavement: (**a**) temperature at middle of pavement (10 cm); (**b**) temperature gradient.

To further elucidate the temperature characteristic of the concrete pavement, a mathematical statistical analysis was conducted on the temperature at the middle of the slab and the temperature gradient. Figure 8 shows the statistical results. From Figure 8, it can be seen that the mean, median, upper and lower limits, as well as the upper and lower quartiles of the temperature of RCP, are all higher than those of OCP. This indicates that, under the same environment, the temperature of RCP is higher than that of OCP. Similarly, the median, lower limits, and lower quartiles of RCP temperature gradients are all smaller than those of OCP temperature gradients (the positive and negative signs of the temperature gradient indicate only the direction of the temperature gradient, not the numerical magnitude). Specifically, the median temperature at the middle of RCP is 1.8 °C higher than that of OCP, and the median RCP temperature gradient is 8.6 °C/m lower than that of OCP. Meanwhile, the amount of negative temperature gradients of RCP and OCP account for 56.88% and 66.85% of the amount of total temperature gradients, respectively. It can be observed that the addition of rubber aggregates can effectively reduce the proportion of negative temperature gradients on concrete pavements, thereby reducing the curling caused by negative temperature gradients. In large-scale concrete pavements, curling is an important factor affecting the size of the pavement, so reducing curling can effectively increase the size of the concrete pavement slab [10,17].

### 4.2. Temperature Strain and Effect

To study the daily variation of temperature strain in concrete pavement, the data (72 h) with the most typical strain variation during the monitoring period were selected as examples for analysis, which include the strains at the middle and bottom of the concrete pavement along the horizontal direction (strains of X-middle, X-bottom, Y-middle, and Y-bottom). To facilitate a comparison between RCP and OCP, the initial data were normalized to zero. Therefore, in the subsequent analysis, the strain values reported for the 72-h period represent the incremental strain relative to the initial moment, rather than absolute strain values. At the same time, in order to describe the daily variation of temperature strain (or stress), the difference between daily maximum and minimum values of strain (or stress) curve was defined as the daily strain (or stress) range.

#### 4.2.1. Temperature Strain

**Figure 9:**
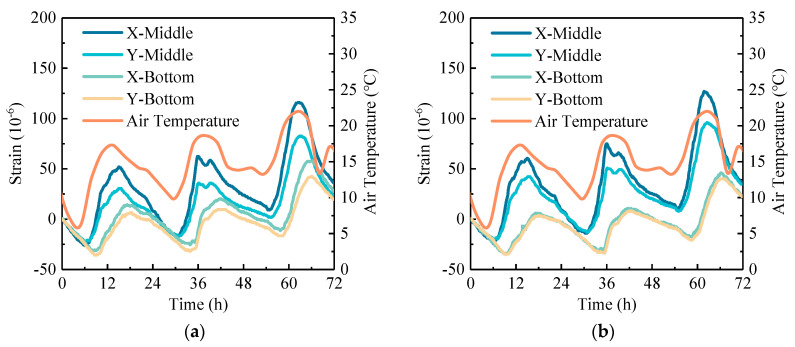
Daily variation curves of temperature strain in concrete pavement: (**a**) OCP and (**b**) RCP.

The daily variation of temperature strain in concrete pavement is shown in Figure 9. It is evident that the temperature strain increases negatively as the air temperature decreases and positively as the air temperature rises. The strain changes in the pavement are periodic but lag behind changes in air temperature, with a more pronounced hysteresis effect observed at the bottom of the pavement. In detail, the phase difference at the middle of the pavement is approximately 1 to 3 h, while the phase difference at the bottom of the pavement is approximately 3 to 5 h. When comparing the strains at the middle and bottom of the pavement, the daily strain range at the middle of the pavement is greater than that at the bottom. This is due to the combined effect of decreasing pavement temperature with depth and the additional constraints imposed by the subgrade at the bottom, which result in a reduced daily strain range at the lower layers. A comparison between RCP and OCP shows that the daily strain range at the middle of RCP is greater than that of OCP, while the daily strain range at the bottom is contrary to this. This indicates that, under similar temperature conditions, the middle of RCP experiences larger deformations compared to OCP, while the bottom of RCP deforms less. Moreover, comparing the strains along different axes, it is found that the longitudinal daily strain ranges at the middle of the RCP and OCP are 1.3 to 1.5 times greater than the transverse daily strain range, respectively. This indicates that the temperature-induced strains are basically consistent in the longitudinal and transverse directions across different pavement materials.

#### 4.2.2. Temperature–Strain Effect

During the monitoring, the influence of rainfall and traffic is minimal, so the strain of pavement is mainly caused by temperature. Meanwhile, temperature deformation mainly includes expansion and contraction from uniform temperature changes and curling deformation from non-uniform temperature.

(1) Temperature Expansion–contraction Effect

**Figure 10:**
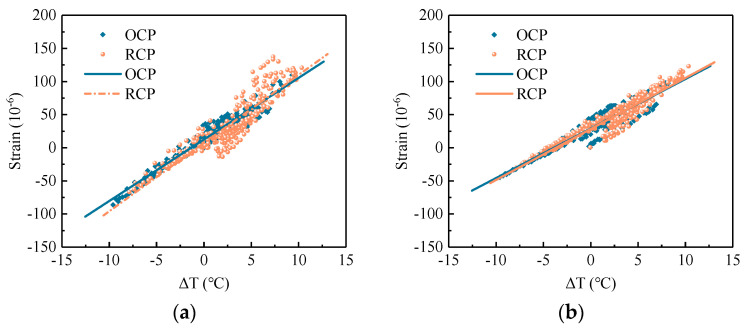
Temperature expansion–contraction effects of concrete pavement: (**a**) *x*-axis; (**b**) *y*-axis.

The temperature expansion–contraction effect of the pavement refers to the relationship between the strain changes and temperature changes at the middle of the pavement. In order to standardize the analysis and eliminate the influence of starting point selection in strain analysis, both strains and temperatures were expressed as increments. Figure 10 shows the temperature expansion–contraction effects of RCP and OCP along different axes. Table 6 shows the fitting parameters derived from the temperature expansion–contraction effect analysis of concrete pavement. From Figure 10 and Table 6, it can be observed that the temperature–strain distribution of RCP along the *x*-axis is significantly broader than that of OCP. Additionally, the slope of the fitting line between temperature and strain for RCP is also greater than that for OCP. In other words, within the same temperature rise range, RCP exhibits a more pronounced temperature–strain hysteresis effect and a larger strain induced by temperature along the *x*-axis. Specifically, for a 10 °C temperature rise, the strain increment along the *x*-axis for RCP is 1.11 times that for OCP. In contrast, along the *y*-axis, the temperature–strain distribution widths and the slopes of fitting line of RCP and OCP are similar. This indicates that the temperature–strain hysteresis effect along the *y*-axis is similar for both RCP and OCP, and the strain response to temperature range is also similar.

(2) Temperature Curling Effect

Under the influence of the non-uniform temperature fields, the strains along the thickness direction of the pavement are distributed unevenly. Depending on whether the temperature gradient is negative or positive, the pavement slab exhibits upward or downward curling. This curvature effect is most pronounced at the center of the pavement slab. According to the plate shell theory, the following assumptions was formulated to calculate the curvature of pavement: (1) the concrete pavement slab is homogeneous, elastic, and isotropic; (2) the vertical stress and strain are zero; (3) temperature and shrinkage strains vary only in the vertical direction; (4) the cross-section remains planar after bending; and (5) the deflection of the pavement slab is small relative to its dimensions. Based on these assumptions, the curvature due to curling can be calculated using Equation (4), whose strains are obtained from monitoring [31,32]. A larger absolute value of curvature indicates more severe pavement warping. The positive and negative values of the curvature reflect the curling direction of the pavement, with positive values indicating upward curling and negative values indicating downward curling.

(4) $$\rho =-\frac{\varepsilon_{t}-\varepsilon_{b}}{h(1+\varepsilon_{t}+\varepsilon_{b})}$$

where *ρ* is the curvature of pavement, m^−1^; *ε*_t_ and *ε*_b_ are the strains at middle and bottom of pavement, 10^−6^; *h* is the distance between strain sensors at the middle and bottom of pavement, m.

**Figure 11:**
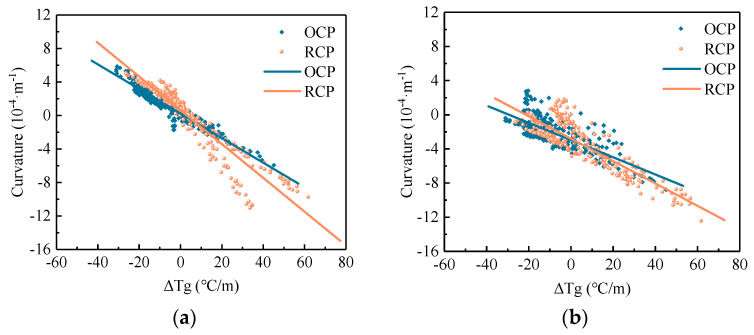
Temperature curling effects of concrete pavement: (**a**) *x*-axis and (**b**) *y*-axis.

Figure 11 shows the temperature curling effects along different axes for RCP and OCP, and Table 7 provides the fitting parameters for the temperature curling effects of concrete pavement. The analysis reveals that the curvature of the pavement is generally negative under positive temperature gradients and positive under negative temperature gradients. Moreover, the curvature magnitude increases with the increase in temperature gradients. Furthermore, along the *x* or *y*-axis, the absolute values of the fitting slopes for the temperature curling effects of RCP are greater than those of OCP. Specifically, whenever the temperature gradient increases by 10 °C/m, the curvature of RCP increases by 2.02 × 10^−5^ and 1.31 × 10^−5^ along the *x* and *y*-axes, respectively, whereas for OCP, it increases by 1.47 × 10^−5^ and 1.01 × 10^−5^ along the *x* and *y*-axes, respectively. The increases in curvature along the *x* and *y*-axes of RCP are 1.37 and 1.30 times those of OCP along the *x* and *y*-axes, respectively. This indicates that, under an equivalent temperature gradient, RCP exhibits greater curvature and stronger curling effects compared to OCP. Therefore, particular attention should be paid to large-scale RCP designs.

### 4.3. Temperature Stress

In order to compare the stress difference between RCP and OCP, the structural stresses in the *x* and *y*-axes of the concrete pavement can be calculated using Equations (5) and (6) based on the elastic plate theory, respectively [33].

(5) $$\sigma_{x}=\frac{E}{1-\mu^{2}}\left(\varepsilon_{x}+\mu \varepsilon_{y}\right)-\frac{E\alpha T_{z}}{1-\mu }$$

(6) $$\sigma_{y}=\frac{E}{1-\mu^{2}}\left(\varepsilon_{y}+\mu \varepsilon_{x}\right)-\frac{E\alpha T_{z}}{1-\mu }$$

where *σ_x_* and *σ_y_* are the temperature stress in the *x* and *y*-axes, respectively, MPa; *E* is the elastic modulus, MPa; *ε*_x_ and *ε*_y_ are the temperature strain in the *x* and *y*-axes, respectively, με; *μ* is poisson’s ratio; *α* is the coefficient of thermal expansion, m·°C^−1^; *T*_z_ is the temperature at the calculation point, °C.

**Figure 12:**
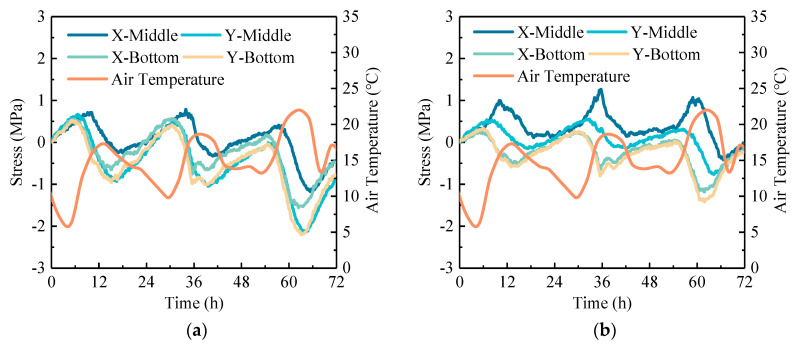
Daily variation curve of temperature stress of concrete pavement: (**a**) OCP and (**b**) RCP.

The temperature stresses were calculated based on the selected temperature strains. For these calculations, the elastic modulus values are taken as listed in Table 3, and the Poisson’s ratio was set to 0.2 for all cases. Existing studies have shown that rubber aggregate has little effect on the thermal expansion coefficient of concrete. According to the existing research results, the thermal expansion coefficient is set at 9.6 × 10^−6^ for rubberized cement concrete and 10.2 × 10^−6^ for ordinary cement concrete [34]. Figure 12 presents the stress increment caused by temperature changes relative to the initial moment. The data reveal that the stress in the pavement also presents cyclical changes over time, similar to air temperature. Analyzing the daily stress range of pavement, it is found that, under the same temperature change, the daily stress range at the bottom of the pavement is slightly greater than that at the middle, which is different from the daily strain range where the middle is greater than the bottom. Meanwhile, the daily stress range of OCP is greater than that of RCP. In detail, along the *x*-axis, the daily stress range of RCP is 0.73 times that of OCP; along the *y*-axis, the daily stress range of RCP is 0.64 times that of OCP. Thus, under the same conditions, the daily strain range of RCP is greater than that of OCP, but its daily temperature stress range is smaller than that of OCP, allowing RCP to withstand greater temperature loads.

## 5. Conclusions

This study established a pavement response monitoring system using fiber Bragg grating testing technology, and then investigated the temperature field and strain distribution characteristics, analyzed the relationship between temperature and strain, and calculated the temperature stresses in the concrete pavement. The following conclusions were drawn:

(1) The daily temperature variation curve of the concrete pavement exhibits significant asymmetry, with the heating phase accounting for only one-third. The addition of rubber particles alters the temperature distribution of concrete pavement. Particularly during the cooling stage, RCP shows a significant “spindle-shaped” characteristic, where the temperature at the middle is higher than those at the top and bottom of the RCP. The positive temperature gradient of RCP is greater than that of OCP, its negative temperature gradient is smaller than that of OCP, and its median temperature gradient is 8.6 °C/m lower than that of OCP. Additionally, the proportions of negative temperature gradients of RCP and OCP are 56.88% and 66.85%, respectively.
(2) The daily strain range at the middle of concrete pavement is greater than that at the bottom, and the addition of rubber particles will increase this phenomenon. Meanwhile, the addition of rubber aggregates makes the temperature–strain hysteresis effect more pronounced, makes the temperature–strain change along the *x*-axis large, and increases the curling curvature of concrete pavement slab. When the temperature rises by 10 °C, the *x*-axis strain of RCP is 1.11 times that of OCP. When the temperature gradient increases by 10 °C/m, the curvature change of RCP is about 1.3 times greater than that of OCP.
(3) The addition of rubber particles reduces the daily stress range at the bottom of the concrete pavement, enabling RCP to bear greater temperature loads. Along the *x*-axis of the pavement, the daily stress range of RCP is 0.73 times that of OCP; along the *y*-axis of the pavement, the daily stress range of RCP is 0.64 times that of OCP.

## Figures and Tables

**Table 1:** Technical characteristics of rubber aggregates.

Properties	Specification
Rubber powder size (mesh)	30–60
Organic matter (%)	67.21
Inorganic content (%)	32.79
Contact angle (°)	0

**Table 2:** Mixing proportions of rubberized cement concrete and ordinary cement concrete (kg/m^3^).

Type	Cement	Water	Rubber Powder	Sand	Aggregate (5–10 mm)	Aggregate (10–19 mm)	Aggregate (19–37.5 mm)	Water-Reducing Agent
Rubberized cement concrete	405	162	110	358	410	410	546	5.7
Ordinary cement concrete	405	162	--	616	410	410	546	5.7

**Table 3:** Properties of rubberized cement concrete and ordinary cement concrete.

Test Items	Test Value		Standard Method (Specification)
	Rubberized Cement Concrete	Ordinary Cement Concrete	
Flexural-tensile strength (MPa)	5.0	6.1	JTG 3420-2020 [29]
Compressive strength (MPa)	36.5	47.9	JTG 3420-2020 [29]
Elasticity modulus (MPa)	36,700	44,000	JTG 3420-2020 [29]

**Table 4:** Technical parameters of temperature and strain sensor.

Parameter Type	Temperature Sensor	Strain Sensor
Measuring range	−40–200 °C	−1500 × 10^−6^–1000 × 10^−6^
Distinguishability	0.1%F.S	0.1%F.S
Reflectivity	≥90%	≥90%
Operating wavelength range *	1510–1590 nm	1510–1590 nm

* The center wavelength of each sensor is fixed, but this range refers to the center wavelength range of a group of sensors connected in series.

**Table 5:** Technical parameters of fiber Bragg grating demodulator.

Properties	Specification
Wavelength resolution	1 pm
Wavelength accuracy	±1 pm
Sampling frequency	1−100 Hz
Dynamic range	50 dB
Wavelength range	1510–1590 nm

**Table 6:** Fitting parameters of temperature–strain expansion effects of concrete pavement.

Axis Type	OCP			RCP		
	Slope	Intercept	R^2^	Slope	Intercept	R^2^
*x*-axis	9.29	11.44	0.85	10.29	5.93	0.84
*y*-axis	7.47	28.03	0.94	7.70	27.37	0.95

**Table 7:** Fitting parameters of temperature curling effects of concrete pavement.

Axis Type	OCP			RCP		
	Slope	Intercept	R^2^	Slope	Intercept	R^2^
*x*-axis	−1.47 × 10^−5^	2.26 × 10^−5^	0.93	−2.02 × 10^−5^	6.23 × 10^−5^	0.90
*y*-axis	−1.01 × 10^−5^	−2.99 × 10^−4^	0.57	−1.31 × 10^−5^	−2.83 × 10^−4^	0.74

## Data Availability

The datasets used or analyzed during the current study are available from the corresponding author on reasonable request.

## References

[1] Yuan J., Li W., Li Y., Ma L., Zhang J. (2021). Fatigue models for airfield concrete pavement: Literature review and discussion. Materials.

[2] Zhang J., Zhang G., Wang Y., Yuan J. (2022). Mechanical behavior of doweled joints in concrete pavements: A review. J. Transp. Eng. Part B Pavements.

[3] Yao A., Ding H., Zhang X., Hu Z., Hao R., Yang T. (2018). Optimum design and performance of porous concrete for heavy-load traffic pavement in cold and heavy rainfall region of NE China. Adv. Mater. Sci. Eng..

[4] Nam B.H. (2022). In-situ super accelerated pavement test for the fatigue evaluation of in-service airfield rigid pavement-A case study at Mecham Airport. Constr. Build. Mater..

[5] Suparp S., Ali N., Al Zand A.W., Chaiyasarn K., Rashid M.U., Yooprasertchai E., Hussain Q., Joyklad P. (2022). Axial load enhancement of lightweight aggregate concrete (LAC) using environmentally sustainable composites. Buildings.

[6] Wei Y., Gao X., Wang F., Zhong Y. (2019). Nonlinear strain distribution in a field-instrumented concrete pavement slab in response to environmental effects. Road Mater. Pavement Des..

[7] Zhao H., Ma L., Zhang J. (2018). Effects of temperature variations on the deflections of airfield jointed plain concrete pavements. Int. J. Transp. Sci. Technol..

[8] Nantasai B., Nassiri S. (2019). Winter temperature prediction for near-surface depth of pervious concrete pavement. Int. J. Pavement Eng..

[9] Liang R.Y., Niu Y.Z. (1998). Temperature and curling stress in concrete pavements: Analytical solutions. J. Transp. Eng..

[10] Zhang G., Zhang J., Yuan J., Ye S. (2023). Horizontal distribution of temperature effect in rubberized concrete pavement: A case study. Buildings.

[11] Quan L., Tian B., Chen L. (2017). Experimental investigation of built-in curling evolution in full scale concrete pavement. J. Highw. Transp. Res. Dev..

[12] Liao W., Zhuang Y., Zeng C., Deng W., Huang J., Ma H. (2020). Fiber optic sensors enabled monitoring of thermal curling of concrete pavement slab: Temperature, strain and inclination. Measurement.

[13] Guo S., Dai Q., Si R., Sun X., Lu C. (2017). Evaluation of properties and performance of rubber-modified concrete for recycling of waste scrap tire. J. Clean. Prod..

[14] Roychand R., Gravina R.J., Zhuge Y., Ma X., Youssf O., Mills J.E. (2020). A comprehensive review on the mechanical properties of waste tire rubber concrete. Constr. Build. Mater..

[15] Mubaraki M., Abd-Elhady A., Sallam H. (2013). Mixed mode fracture toughness of recycled tire rubber-filled concrete for airfield rigid pavements. Int. J. Pavement Res. Technol..

[16] Fiore A., Marano G.C., Marti C., Molfetta M. (2014). On the fresh/hardened properties of cement composites incorporating rubber particles from recycled tires. Adv. Civ. Eng..

[17] Zhu H., Jiao X., Li J., Liao M., Tang B. (2019). Analysis and calculation of ratio of length to thickness of crumb rubber concrete airport pavement slab. J. Shijiazhuang Tiedao Univ. (Nat. Sci. Ed.).

[18] Zhang G., Yuan J., Zhang J., Xu W. (2023). Variation characteristics of temperature field of rubber concrete pavement. J. Chang’an Univ. (Nat. Sci. Ed.).

[19] Sukontasukkul P. (2009). Use of crumb rubber to improve thermal and sound properties of pre-cast concrete panel. Constr. Build. Mater..

[20] Ocholi A., Ejeh S.P., Yinka S.M. (2014). An investigation into the thermal performance of rubber-concrete. Acad. J. Interdiscip. Stud..

[21] Chen J., Wang H., Xie P. (2019). Pavement temperature prediction: Theoretical models and critical affecting factors. Appl. Therm. Eng..

[22] Xue G., Sun L., Pei Z. (2019). Analysis of structural mechanics response of rubber concrete pavement under temperature gradient. J. Inn. Mong. Univ. Sci. Technol..

[23] Ferretti E. (2012). Waste tire rubberized concrete plates for airport pavements: Stress and strain profiles in time and space domains. Comput. Mater. Contin..

[24] Rao S., Roesler J.R. (2005). Characterizing effective built-in curling from concrete pavement field measurements. J. Transp. Eng..

[25] Oh H.J., Cho Y.K., Seo Y., Kim S.M. (2016). Experimental analysis of curling behavior of continuously reinforced concrete pavement. Constr. Build. Mater..

[26] Wang J., Han Y., Cao Z., Xu X., Zhang J., Xiao F. (2023). Applications of optical fiber sensor in pavement Engineering: A review. Constr. Build. Mater..

[27] Zhou Z., Liu W., Huang Y., Wang H., He J., Huang M., Ou J. (2012). Optical fiber Bragg grating sensor assembly for 3D strain monitoring and its case study in highway pavement. Mech. Syst. Signal Process..

[28] Kashaganova G., Kozbakova A., Kartbayev T., Balbayev G., Togzhanova K., Alimseitova Z., Orazaliyeva S. (2023). Research of a fiber sensor based on fiber Bragg grating for road surface monitoring. Electronics.

[29] (2020). Testing Methods of Cement and Concrete for Highway Engineering.

[30] Janssen D.J., Snyder M.B. (2000). Temperature-moment concept for evaluating pavement temperature data. J. Infrastruct. Syst..

[31] Ling J., Liu H., Shi R., Yang F., Tang L. (2022). Effect of diurnal temperature variations on airport cement pavement. J. Tongji Univ. (Nat. Sci.).

[32] Asbahan R.E., Vandenbossche J.M. (2011). Effects of temperature and moisture gradients on slab deformation for jointed plain concrete pavements. J. Transp. Eng..

[33] Tian B., Quan L., Niu K. (2014). Structural experiment and theoretical analysis of thermal curling in JPCP with different base types. China J. Highw. Transp..

[34] Xu J., Hong J., Wan Y., Liu J. (2009). Influence of rubber on thermal performance of concrete. Concrete.

